# Non-driver mutations landscape in different stages of primary myelofibrosis determined *ASXL1* mutations play a critical role in disease progression

**DOI:** 10.1038/s41408-023-00829-3

**Published:** 2023-04-20

**Authors:** Xin Yan, Zefeng Xu, Peihong Zhang, Qi Sun, Yujiao Jia, Tiejun Qin, Shiqiang Qu, Lijuan Pan, Zhanqi Li, Jinqin Liu, Zhen Song, Qingyan Gao, Meng Jiao, Jingye Gong, Huijun Wang, Bing Li, Zhijian Xiao

**Affiliations:** 1 State Key Laboratory of Experimental Hematology, National Clinical Research Center for Blood Diseases, Haihe Laboratory of Cell Ecosystem, Institute of Hematology & Blood Diseases Hospital, Chinese Academy of Medical Sciences & Peking Union Medical College, Tianjin, 300020 China; 2 Tianjin Institutes of Health Science, Tianjin, 301600 China; 3 MDS and MPN Centre, Institute of Hematology & Blood Diseases Hospital, Chinese Academy of Medical Sciences & Peking Union Medical College, Tianjin, 300020 China; 4 Hematologic Pathology Centre, Institute of Hematology & Blood Diseases Hospital, Chinese Academy of Medical Sciences & Peking Union Medical College, Tianjin, 300020 China; 5 Centre for Information and Resources, Institute of Hematology & Blood Diseases Hospital, Chinese Academy of Medical Sciences & Peking Union Medical College, Tianjin, 300020 China

**Keywords:** Diseases, Myeloproliferative disease

Dear Editor,

In primary myelofibrosis (PMF), there is a stepwise evolution from an initial prefibrotic (Pre)/early stage, characterized by hypercellular bone marrow with absent or minimal reticulin fibrosis, to an overt fibrotic (Overt) stage with marked reticulin or collagen fibrosis in the bone marrow [1] and 5–30% of patients develop to blast phase [2–6]. Although gene mutations were confirmed as the important prognostic factor in PMF [7–12], whether or not these gene mutations can predict fibrosis progression and leukemic transformation is still unclear. The aim of this study was to explore if some non-driver mutations play a key role in disease progression based on the mutational landscape in the progression of PMF. Through analyzing the non-driver mutations in different stages of PMF patients, we found that *ASXL1* mutations were significantly associated with both the exacerbation of fibrosis and the leukemic transformation.

According to the World Health Organization (WHO) 2016 classification [13], 258 consecutive PMF patients from July 7, 2015 to December 23, 2021 were enrolled. All cases were reviewed by two experienced pathologists (PZ, QS) to confirm the diagnosis of PMF, evaluate disease progression and exclude post-PV/ET MF according to the WHO 2016 classification of MPN [13]. A total of 275 samples from different stages of disease were included in the analysis: 69 Pre-PMF, 161 Overt-PMF and 45 PMF-AP/BP (details in Supplementary Fig. 1). At least two-time points serial samples from 17 patients during disease progression (4 patients progressed from Pre-PMF to Overt-PMF and 13 patients progressed from Overt-PMF to PMF-AP/BP) were sequenced. The median interval from PMF diagnosis to AP/BP transformation was 28.5 (1–144) months. Patient samples were obtained with written informed consent by the Declaration of Helsinki, and the study was approved by the human research ethics committee at the Institute of Hematology and Blood Disease Hospital, Chinese Academy of Medical Sciences (CAMS) and Peking Union Medical Collage (PUMC) according to the guidelines of the Declaration of Helsinki.

Twenty-seven genes (Supplementary Table 1) were employed to investigate the frequency and enrichments of non-driver mutations in different stages of PMF. High molecular risk (HMR) mutations were considered as mutations in any one of the six genes: *ASXL1, EZH2, IDH1, IDH2, SRSF2 and U2AF1Q157* [11]. More detailed information is described in the Supplementary Methods.

Comparison of the baseline clinical and laboratory characteristics of patients classified in different stages are summarized in Supplementary Table 4. Driver mutations were distributed as follows: *JAK2*^V617F^ (55.8%, *n* = 144), *CALR* Exon9 (24%, *n* = 62), *MPL*^W515^ (3.5%, *n* = 9), double mutations (*CALR* Exon9 and *MPL*^W515^) in 1 patient (0.4%). Forty two (16.3%) patients did not have any driver mutations (triple-negative). Only the variant allele frequency(VAF) of *JAK2*^V617F^ had a significant increment between Pre-PMF and PMF-AP/BP patients (median of VAF: 38.3% vs. 50.9%, *P* = 0.031), but no difference between Overt-PMF and PMF-AP/BP (median of VAF: 45.4% vs.50.9%, *P* = 0.333) (Supplementary Fig. 2A–C). The distribution and the number of non-driver mutations in different stages of PMF were shown in Supplementary Fig. 2D–G.

Mutations in the *ASXL1* (31.1% vs. 10.1%, *P* = 0.001) and *U2AF1* (13.7% vs. 1.4%, *P* = 0.003) were more frequent in Overt-PMF compared with Pre-PMF (Fig. 1A). Moreover, mutations in *ASXL1* (51.1% vs. 31.1%, *P* = 0.013), *SRSF2* (26.7% vs. 11.2%, *P* = 0.009), *RUNX1* (22.2% vs. 5.6%, *P* = 0.001), *SETBP1* (17.8% vs. 7.5%, *P* = 0.039), *NRAS* (15.6% vs. 3.1%, *P* = 0.002) and *EZH2* (13.3% vs. 5%, *P* = 0.049) were significantly more frequent in PMF-AP/BP compared with Overt-PMF (Fig. 1B). Consistent with prior studies [14, 15], *ASXL1* mutations were most common in PMF and frameshift and nonsense mutations are the major *ASXL1* mutation types (Supplementary Fig. 3). From the whole course of the disease, the frequency of *ASXL1* mutations significantly increased during the progression (10.1% vs. 31.1% vs. 51.1%) (Fig. 1C). However, there was no significant change in the VAF of *ASXL1* mutations in different stages (Median of VAF: 42.1% vs. 30.6% vs. 37.8%) (Supplementary Fig. 2H). In addition, the frequency of HMR mutations also significantly increased during the disease progression (14.5% vs. 45.3% vs.73.3%) (Supplementary Fig. 2I) as expected.

**Fig. 1:**
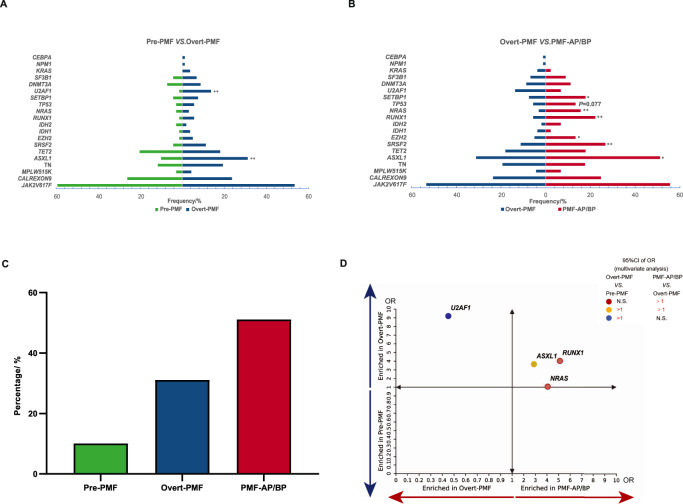
The association between non-driver mutation and different stages of PMF. Comparison of mutation frequency in Pre-PMF vs. Overt-PMF (**A**) and Overt-PMF vs. PMF-AP/BP patients (**B**). **C** The frequency of *ASXL1* mutations in different stages of PMF. **D** Enrichment of non-driver mutations in PMF-AP/BP and Overt-PMF relative to Overt-PMF and Pre-PMF, respectively. Enrichment is expressed as an odds ratio (OR) of mutation rates in PMF-AP/BP vs. Overt-PMF on the x-axis and Overt-PMF vs. Pre-PMF on the y-axis. Mutations showing significant enrichment in either comparison are indicated by colors according to OR 95% CI limits being above (if OR > 1) or below (if OR < 1). *P < 0 .05; **P < 0.01; ***P < 0.001.

To clarify the relationship between non-driver mutations and disease progression, we looked at the enrichments of major non-driver mutations in different stages of PMF.

In univariate comparison, *ASXL1* (OR = 3.99, 95% CI 1.71–9.33; *P* = 0.001) and *U2AF1* (OR = 10.76, 95% CI 1.42–81.53; *P* = 0.005) mutations were significantly enriched in Overt-PMF compared to Pre-PMF. Mutations in five genes, including *ASXL1*(OR = 2.32, 95% CI 1.18–4.55; *P* = 0.013), *SETBP1* (OR = 2.69, 95% CI 1.02–7.64; *P* = 0.039), *RUNX1* (OR = 4.83, 95% CI 1.82–12.76; *P* = 0.001), *NRAS* (OR = 5.75, 95% CI 1.73–19.10; *P* = 0.002) and *SRSF2* (OR = 2.89, 95% CI 1.27–6.58; *P* = 0.009) were significantly enriched in PMF-AP/BP compared to Overt-PMF (Supplementary Fig. 2J and Supplementary Table 5).

To exclude the effects of the co-occurrence of non-driver mutations that might confound the result, we performed a multivariate analysis. In the comparison between Overt-PMF and Pre-PMF, *ASXL1* (OR = 3.65, 95% CI 1.54–8.62; *P* = 0.003) and *U2AF1* (OR = 9.21, 95% CI 1.20–70.69; *P* = 0.033) mutations were still significantly enriched in Overt-PMF. Meanwhile, in the comparison between PMF-AP/BP and Overt-PMF, only *RUNX1* (OR = 5.11, 95% CI 1.66–15.74; *P* = 0.004), *NRAS*(OR = 4.06, 95% CI 1.05–15.60; *P* = 0.042) and *ASXL1* (OR = 2.88, 95% CI 1.36–6.10; *P* = 0.006) mutations were strongly enriched in PMF-AP/BP (Fig. 1D and Supplementary Table 5). These results indicate that *ASXL1* mutations might play a critical role in myelofibrosis progression and blast phase evolution during the whole course of PMF progression.

As mentioned above, *ASXL1* mutations play a critical role both in the progression from Pre-PMF to Overt-PMF and through Overt-PMF to PMF-AP/BP. To evaluate the status of *ASXL1* mutations in different stages, we analyzed 17 patients who had at least two-time points serial samples during disease progression (Supplementary Table 6). Firstly, to explore how *ASXL1* mutations promote the progression from the chronic stage to the AP/BP stage, we analyzed clonal evolution in serial samples collected from Overt-PMF to PMF-AP/BP. Excepting newly acquired *ASXL1* mutations at the AP/BP stage, all *ASXL1* mutations that occurred in the Overt-PMF phase kept relatively stable with less than 10% of fluctuation (Fig. 2A). Contrary to *ASXL1*, *RUNX1* mutations (4 in 5, 80%), *RAS* pathway mutations (7 in 8, 87.5%) and *TP53* mutations (1 in 1,100%) were more likely to be freshly acquired during the transformation from Overt-PMF to PMF-AP/BP (Fig. 2B and Supplementary Fig. 4A and B). Interestingly, *SRSF2* mutations tended to decrease clone size during disease progression (Supplementary Fig. 4C). Then, to answer the question that why AP/BP transformation did not accompany with obvious *ASXL1* mutations expansion, we analyzed the co-mutations in PMF-AP/BP patients who had *ASXL1* mutations at the chronic stage. We found that *ASXL1* mutations were more likely to co-occur with *RAS* pathway mutations (*NRAS* 20% and *NF1* 50%) and *ETV6* (50%) (Fig. 2C). Data of serial samples from chronic and AP/BP stages shed the light on that different non-driver mutations should play different roles in the AP/BP transformation: 1. *ASXL1* mutations are not a direct event for leukemogenesis but accelerate the possibility of accumulation of direct leukemogenic factors, such as *RAS* pathway mutations. 2. *RUNX1* mutations are direct and independent factors in AP/BP transformation. Moreover, *ASXL1* mutations even cannot be significantly removed by hypomethylating agent (HMA) therapy in 3 PMF-AP/BP patients (Fig. 2D), but co-occurred *RUNX1* and *SETBP1* mutations burden were obviously decreased (Supplementary Fig. 4D).

**Fig. 2:**
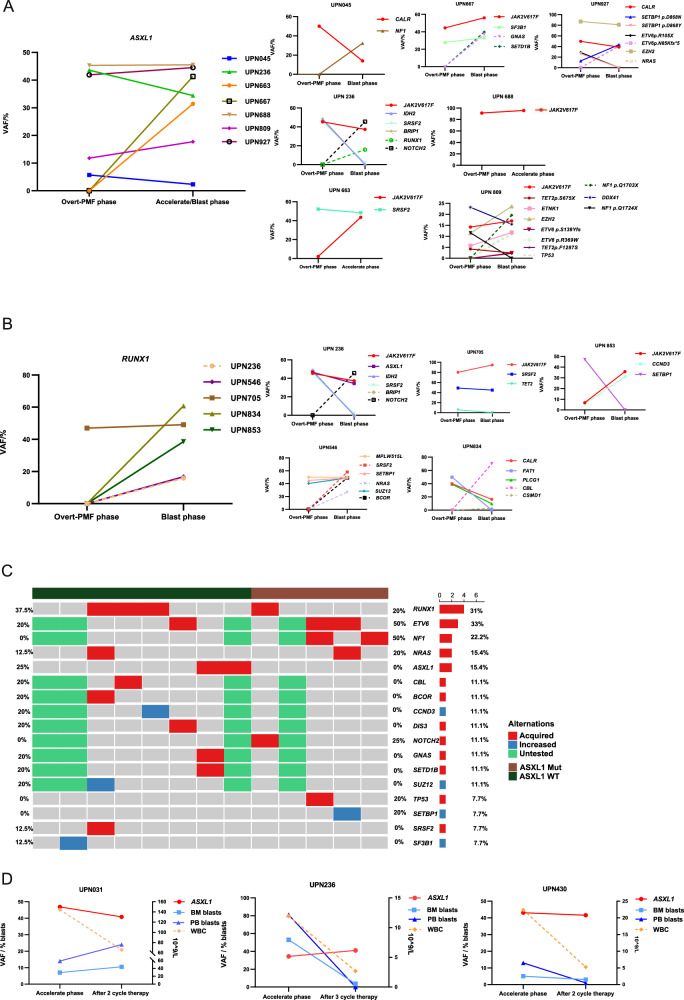
Dynamics of mutations in the AP/BP transformation in PMF. **A** VAF of mutations in 7 paired serial samples from PMF patients with *ASXL1* mutation. **B** VAF of mutations in 5 paired serial samples from PMF patients with *RUNX1* mutation. In (**A**) and (**B**), the left panel showed the VAF of *ASXL1* or *RUNX1* mutation and the right panel showed the VAF of other mutations in each patient. **C** The number and proportion of patients with newly acquired and VAF-increasing mutations during the AP/BP transformation were indicated in the hot plot. Newly acquired mutations were depicted in red and VAF-increasing mutations (>10%) were depicted in blue, and untested genes were depicted in green. Patients with and without *ASXL1* mutation in Overt-PMF were labeled in brown and blackish green, respectively. **D** VAF of *ASXL1* mutation, percentage of peripheral blood/bone marrow blasts as well as white blood cell counts before and after HMA therapy in PMF-AP/BP.

In summary, our study provides a clue that *ASXL1* mutations may play a critical role in the whole course of PMF, aggravating myelofibrosis and conferring the tractive force for leukemia transformation. Due to the high frequency and critical role of *ASXL1* mutations in PMF, *ASXL1* could potentially act as a therapeutic intervention point. Treatments targeting the functional effect of mutant *ASXL1* in PMF, such as BET (Bromodomain and Extraterminal domain) inhibitors, BAP1(BRCA1-Associated Protein 1) inhibitors, TNFR (Tumor Necrosis Factor Receptor) inhibitors, et al., could be used in the future. Because of a small cohort with limited paired serial samples from a single center, our findings also need to be validated in a large cohort with more paired serial samples.

## Supplementary information

Supplementary information

## Data Availability

The datasets generated during and/or analyzed during the current study are available from the corresponding author on reasonable request.
