# Supplementary material for: Non-driver mutations landscape in different stages of primary myelofibrosis determined ASXL1 mutations play a critical role in disease progression
Source: Blood Cancer J. 2023 Apr 20;13(1):56. doi: 10.1038/s41408-023-00829-3 (PMC10119377; doi:10.1038/s41408-023-00829-3)
Supplement: Supplementary file 1 — Supplementary information [file 41408_2023_829_MOESM1_ESM.pdf]

Supplemental Methods

Next generation sequencing

DNA from bone marrow mononuclear cells was used for NGS as described[1], Sequencing was performed using the Illumina HiSeq platform. 20 samples were sequenced with a 27-gene panel (Supplemental Table 1) and 155 samples were sequenced by using a 141-gene panel (Supplemental Table 2) from July, 2015 to March, 2020. Other 100 samples were sequenced by using a 267-gene panel (Supplemental Table 3) from April, 2020 to December, 2021. If serial samples were sequenced by different panels, only overlapped genes from different panels were used to analyze clonal evolution. Bioinformatics tools were used to call and annotate variants (details in Bioinformatics analysis). Only exonic or splicing mutations (donor and acceptor sites) with a variant allele frequency ≥2% and not described as common polymorphisms (≥1% in the general population) were retained. Variants were finally classified according to their putative pathogenetic effect as pathogenetic, possible pathogenetic, or variant of unknown significance according to standard guidelines (details in Sequence variant annotation). Furthermore, mutations of unknown significance with a frequency ≥0.01% in the general population were considered as rare polymorphisms and were removed. Only pathogenetic and possible pathogenetic variants were kept in the statistical analysis.

Targeted gene sequencing

DNAs from bone marrow aspirates were extracted and prepared for sequencing. Each DNA sample is quantified by agarose gel electrophoresis and Nanodrop (Thermo).

Libraries were prepared using Illumina standard protocol. The amplified DNA was captured with a 27-gene panel(Supplemental Table 1)/ 141-gene panel(Supplemental Table 2) /267-gene panel(Supplemental Table 3), using biotinylated oligo-probes (MyGenostics GenCap Enrichment technologies). The average depth of each NGS panel was 2000x. The capture experiment was conducted according to the manufacturer's protocol. Illumina utilizes a unique "bridged" amplification reaction that occurs on the surface of the flow cell. A flow cell containing millions of unique clusters is loaded into the HiSeq 2000 for automated cycles of extension and imaging. Illumina's Sequencing-by-Synthesis utilizes four proprietary nucleotides possessing reversible fluorophore and termination properties. Each sequencing cycle occurs in the presence of all four nucleotides leading to higher accuracy than methods where only one nucleotide is present in the reaction mix at a time. This cycle is repeated, one base at a time, generating a series of images each representing a single base extension at a specific cluster.

Bioinformatics analysis

For nuclear gene sequencing analysis, high-quality reads were retrieved from raw reads by filtering out the low-quality reads and adaptor sequences using the Solexa QA package and the cutadapt program (http://code.google.com/p/cutadapt/), respectively. SOAPaligner program was then used to align the clean read sequences to the human reference genome (hg19).

After the PCR duplicates were removed by the Picard software, the SNPs were firstly identified using the SOAP snp program (http://soap.genomics.org.cn/soapsnp.html).

Subsequently, we realigned the reads to the reference genome using BWA and identified the insertions or deletions (InDels) using the GATK program (http://www.broadinstitute.org/gsa/wiki/index.php/Home_Page). The identified SNPs and InDels were annotated using the Exome-assistant program (http://122.228.158.106/exomeassistant). Magic Viewer was used to viewing the short read alignment and validate the candidate SNPs and InDels.

Sequence variant annotation

Once low confidence (Depth <5 or VAF <2%) or likely polymorphisms were removed from the dataset, each high confidence variant was annotated by 1000 Genomes, ESP6500, Inhouse, PolyPhen, SIFT and COSMIC to determine pathogenetic.

Each variant was annotated as pathogenetic, possible pathogenetic or unknown in accordance with prior evidence in the literature in respect to the variants or genes associated with the myeloid disease.

a. Pathogenetic

- Known myeloid malignancies related to pathogenetic variants previously reported in the literature;
- Truncating variants (nonsense mutations, essential splice mutations or frameshift indels) in genes implicated in myeloid malignancies through the acquisition of loss of function mutations.

b. Possible pathogenetic

- Previously unreported variants that cluster (±3aa) with known myeloid malignancies related pathogenetic variants in COSMIC.

c. Unknown

- Variants identified outside the range of frequent variants in genes with known myeloid malignancies related pathogenetic variants;
- Variants (even if recurrent) in genes whose role in myeloid malignancies is not yet established.

Statistics:

Mann-Whitney U test and $\chi 2$ /Fisher's exact tests were used to compare continuous and categorical variables. Odds ratio (OR) and *p* values were estimated using Fisher's exact test in pairwise comparisons among genetic features (Performed with SPSS 24.0 software and R Project 16.4.3 software).

Data sets were described with median and range as indicated. Logistic regression models were used to analyze correlations between genetic mutations and disease stages. Variables with a *P*-value <0.05 in univariate analysis were tested in multivariate analysis. All statistical tests were two-sided at the 5% level of significance.

Supplemental Figure 1

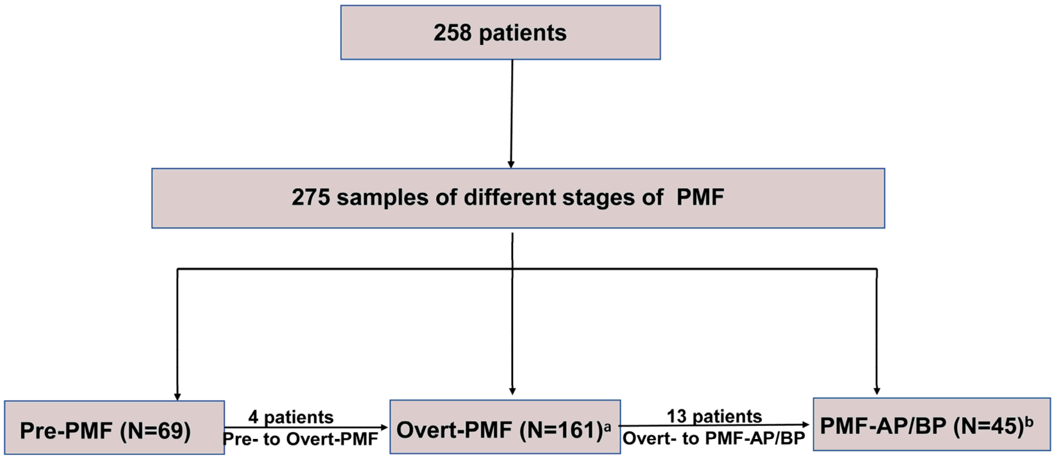

Supplemental Figure 1. Patient enrollment and analysis schema.

a :Overt-PMF samples were collected from 157 freshly diagnosed Overt-PMF patients and 4 patients who progressed to Overt-PMF from the freshly diagnosed Pre-PMF.

b : PMF-AP/BP samples were collected from 32 PMF-AP/BP patients who were diagnosed after July 7, 2015 and 13 patients who progressed to PMF-AP/BP from the freshly diagnosed Overt-PMF.

Supplemental Figure 2

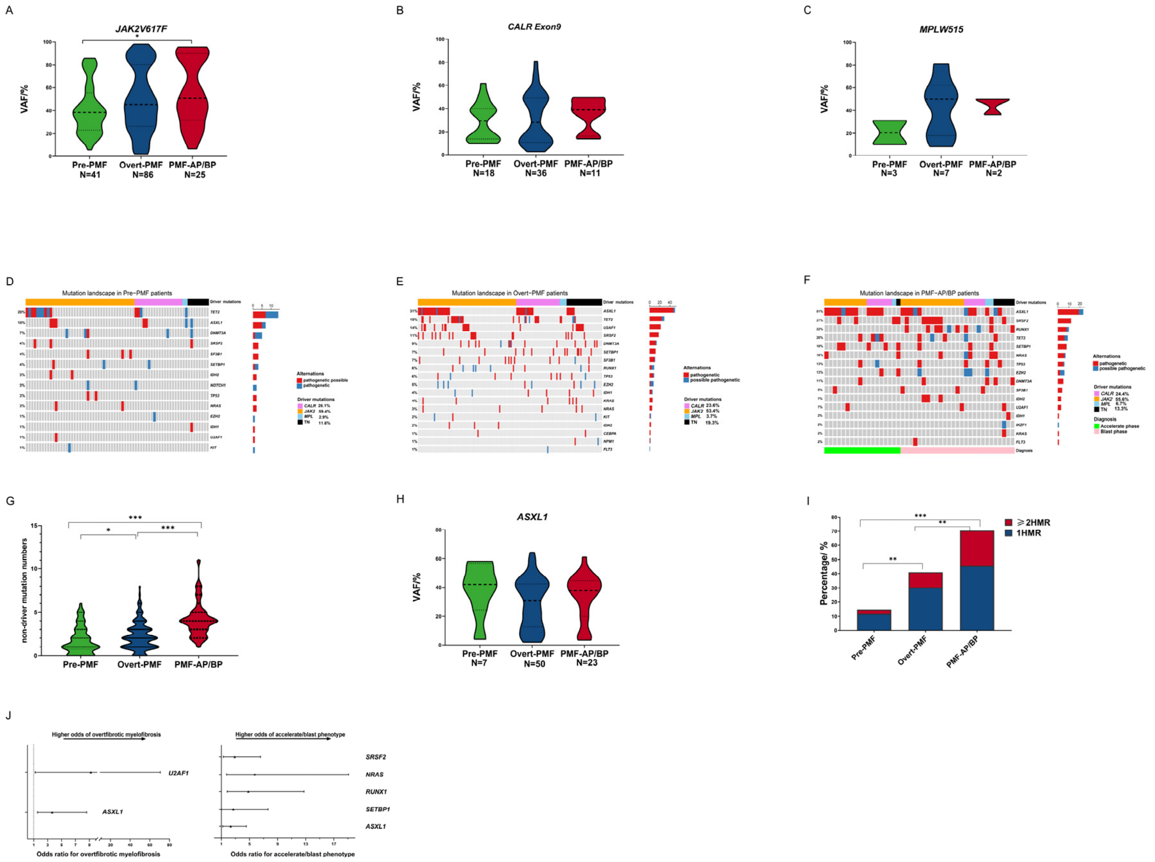

Supplemental Figure 2. Distribution of non-driver mutations in different stages of PMF

(A-C) The variant allele frequency(VAF) of *JAK2V617F*(A), *CALR* Exon9(B) and *MPLW515*(C) mutations in different stages of PMF patients. A total of 65 samples with *CALR* Exon9 mutations were included. 2 samples were excluded in this comparison because of qualitative analysis for *CALR*. (D-F) The distribution and frequency of non-driver mutations which were included in supplemental table 3 in Pre-PMF(D), Overt-PMF(E) and PMF-AP/BP(F) patients. Pathogenetic mutations were depicted in red, and possible pathogenetic mutations were depicted in blue. (G)Numbers of non-driver mutations in different stages of PMF. (H) The VAF of *ASXL1* mutations in different stages of PMF patients. (I)The proportion of HMR mutations in different stages of PMF patients. (J) Forest plots summarized significant associations between non-driver mutations and different stages in univariate analysis.

*$P < 0.05$;**$P < 0.01$; ***$P < 0.001$.

Supplemental Figure 3

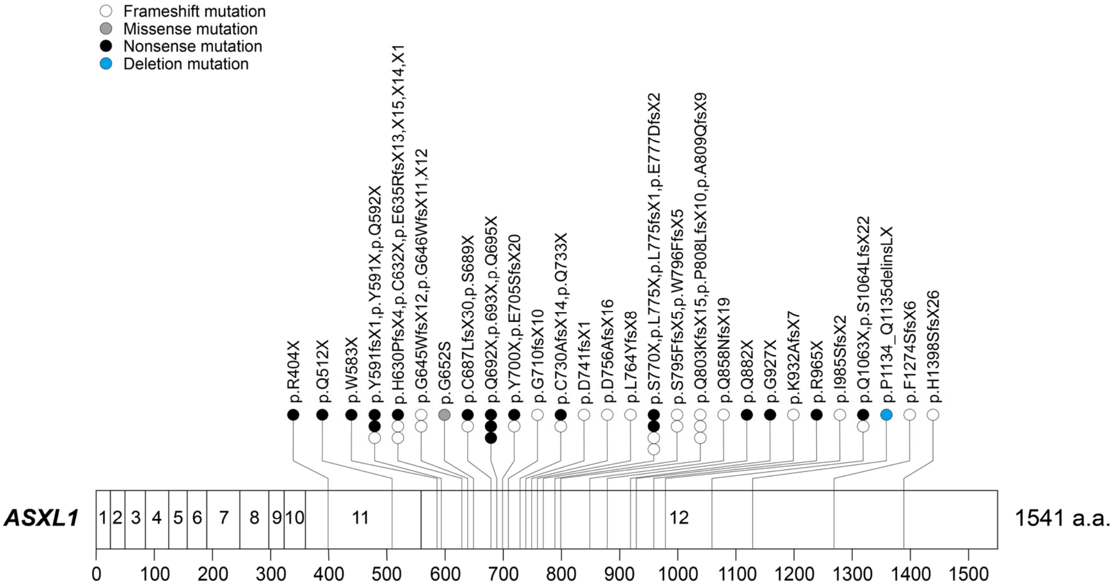

Supplemental Figure 3. Landscape of localizations and mutational types of *ASXL1* mutations in PMF patients. The median coverage of *ASXL1* mutations was 1838× （238× ~ 5980×).

Supplemental Figure 4

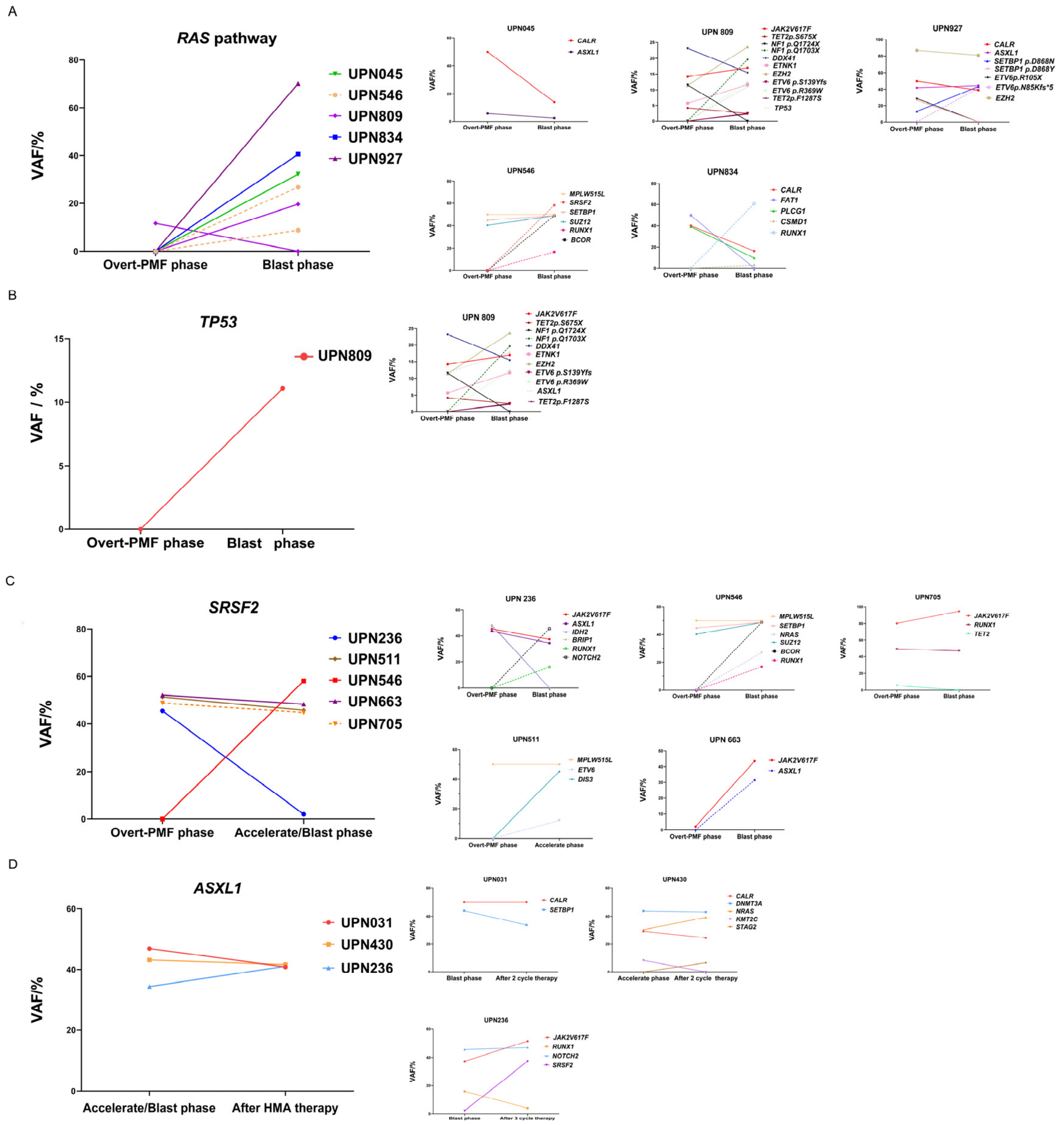

Supplemental Figure 4. Clonal evolution during AP/BP transformation and after HMA therapy based on the analysis of serially collected samples from PMF patients.

VAF of mutations in serial samples from PMF patients with *RAS* pathway mutations (A), *SRSF2* mutations (B) and *TP53* mutations(C). The left panel shows the VAF of *RAS* pathway / *SRSF2* /*TP53* mutations and the right panel shows the VAF of other mutations in each patient with *RAS* pathway/*SRSF2*/*TP53* mutation. (D)VAF of mutations in serial samples (before and after HMA therapy) from PMF-AP/BP patients with *ASXL1* mutation. The left panel shows the VAF of the *ASXL1* mutation and the right panel shows the VAF of other mutations in each patient with *ASXL1* mutation.

Supplemental Table 1. Panel of 27 genes for NGS

ASXL1	*CALR*	*DNMT3A*	*EZH2*	*FLT3*	*IDH1*
IDH2	*JAK2*	*KIT*	*KRAS*	*MPL*	*NRAS*
RUNX1	*TET2*	*TP53*	*BRAF*	*CEBPA*	*CSF3R*
IKZF1	*MYD88*	*NOTCH1*	*NPM1*	*U2AF1*	*SETBP1*
SF3B1	*SRSF2*	*WT1*			

Supplemental Table 2. Panel of 141 genes for NGS

ABL1	*ANKRD26*	*ARID1A*	*ASXL1*	*ASXL2*	*ATG2B*
ATM	*B2M*	*BCL2*	*BCL6*	*BCOR*	*BCORL1*
BIRC3	*BRAF*	*BRINP3*	*BTK*	*CALR*	*CARD11*
CASP8	*CBL*	*CCND1*	*CCND2*	*CCND3*	*CCR4*
CD28	*CD58*	*CD79B*	*CDC25C*	*CDKN1B*	*CDKN2A*
CEBPA	*CNOT3*	*CREBBP*	*CRLF2*	*CSF3R*	*CSNK1A1*
CUX1	*CXCR4*	*DDX3X*	*DDX41*	*DIS3*	*DNM2*
DNMT3A	*DNMT3B*	*EED*	*EGR1*	*EP300*	*ETNK1*
ETV6	*EZH2*	*FAM46C*	*FAT1*	*FBXW7*	*FGFR3*
FLT3	*GATA1*	*GATA2*	*GATA3*	*GNA13*	*ID3*
IDH1	*IDH2*	*IKZF1*	*IL7R*	*IRF4*	*JAK1*
JAK2	*JAK3*	*KDM6A*	*KIT*	*KLF2*	*KMT2A*
KMT2D	*KRAS*	*MAP2K1*	*MAPK1*	*MAX*	*MED12*
MEF2B	*MPL*	*MYC*	*MYD88*	*NF1*	*NOTCH1*
NOTCH2	*NOTCH3*	*NOTCH4*	*NPM1*	*NRAS*	*NT5C2*
PAX5	*PDGFRB*	*PHF6*	*PIGA*	*PLCG1*	*PLCG2*
PPM1D	*PRDM1*	*PRKCB*	*PRPF8*	*PRPS1*	*PTEN*
PTPN11	*RAD21*	*RBBP6*	*RELN*	*RHOA*	*RPL10*
RUNX1	*SETBP1*	*SETD2*	*SF1*	*SF3B1*	*SH2B3*
SMC1A	*SMC3*	*SPEN*	*SRP72*	*SRSF2*	*STAG2*
STAT3	*STAT5B*	*SUZ12*	*TAL1*	*TCF3*	*TERT*
TET2	*TNFAIP3*	*TNFRSF14*	*TP53*	*TPMT*	*TRAF3*
U2AF1	*USP7*	*WHSC1*	*WT1*	*XPO1*	*ZBTB7A*
ZMYM3	*ZNF384*	*ZRSR2*			

Supplemental Table 3. Panel of 267 genes for NGS

ABCB1	*ABL1*	*ANKRD26*	*APC*	*ARID1A*	*ARID1B*
ARID2	*ARID5B*	*ASXL1*	*ASXL2*	*ATG2B*	*ATM*
ATRX	*B2M*	*BACH2*	*BCL10*	*BCL2*	*BCL6*
BCL7A	*BCOR*	*BCORL1*	*BIRC3*	*BLM*	*BPGM*
BRAF	*BRCA1*	*BRCA2*	*BRIP1*	*BTG1*	*BTG2*
BTK	*CALR*	*CARD11*	*CBL*	*CBLB*	*CBLC*
CCND1	*CCND3*	*CCR4*	*CD28*	*CD58*	*CD79A*
CD79B	*CDC25C*	*CDKN1A*	*CDKN1B*	*CDKN2A*	*CDKN2B*
CDKN2C	*CEBPA*	*CHD2*	*CHD8*	*CIITA*	*CNOT3*
CREBBP	*CRLF2*	*CSF1R*	*CSF3R*	*CSMD1*	*CSNK1A1*
CTCF	*CUX1*	*CXCR4*	*CYLD*	*DDX3X*	*DDX41*
DIS3	*DKC1*	*DNM2*	*DNMT3A*	*DNMT3B*	*DTX1*
DUSP2	*EBF1*	*EED*	*EGFR*	*EGLN1*	*EGR1*
ELANE	*EP300*	*EPHA7*	*EPOR*	*ETNK1*	*ETV6*
EZH2	*FAM46C*	*FAS*	*FAT1*	*FAT4*	*FBXO11*
FBXW7	*FGFR3*	*FLT3*	*FOXO1*	*FYN*	*GAB2*
GATA1	*GATA2*	*GATA3*	*GFI1*	*GNA13*	*GNAI2*
GNAS	*GNB1*	*GSKIP*	*H1-2*	*H1-3*	*H1-4*
H1-5	*HAX1*	*HLA-A*	*HLA-C*	*HLA-DMB*	*HNRNPK*
HRAS	*HUWE1*	*HVCN1*	*ID3*	*IDH1*	*IDH2*
IGLL5	*IKZF1*	*IKZF2*	*IKZF3*	*IL7R*	*IRF2BP2*
IRF4	*IRF8*	*ITPKB*	*JAK1*	*JAK2*	*JAK3*
JUNB	*KDM6A*	*KIT*	*KLF2*	*KLHL6*	*KMT2A*
KMT2B	*KMT2C*	*KMT2D*	*KRAS*	*KRT20*	*LCOR*
LMO2	*LTB*	*LYN*	*MAP2K1*	*MAPK1*	*MAX*
MCL1	*MED12*	*MEF2B*	*MFHAS1*	*MPL*	*MTOR*
MYC	*MYCN*	*MYD88*	*MYOM2*	*NF1*	*NFE2*
NFKBIA	*NFKBIE*	*NOTCH1*	*NOTCH2*	*NOTCH3*	*NOTCH4*
NPM1	*NRAS*	*NT5C2*	*P2RY8*	*PALB2*	*PAX5*
PDGFRA	*PDGFRB*	*PDS5B*	*PHF6*	*PIGA*	*PIK3CA*
PIK3CD	*PIK3R1*	*PIM1*	*PIM2*	*PLCG1*	*PLCG2*
POT1	*PPM1D*	*PRDM1*	*PRF1*	*PRKCB*	*PRKD2*
PRKDC	*PRPF8*	*PRPS1*	*PSMB5*	*PTEN*	*PTPN1*
PTPN11	*PTPRD*	*RAD21*	*RASA2*	*RB1*	*RBBP6*
RELN	*RHOA*	*RPL10*	*RRAGC*	*RUNX1*	*SAMHD1*
SBDS	*SETBP1*	*SETD1B*	*SETD2*	*SETDB1*	*SF1*
SF3B1	*SGK1*	*SH2B3*	*SH2D1A*	*SMARCA4*	*SMC1A*
SMC3	*SMO*	*SOCS1*	*SP140*	*SPEN*	*SRP72*
SRSF2	*STAG2*	*STAT3*	*STAT5B*	*STAT6*	*SUFU*
SUZ12	*SYK*	*TAL1*	*TBL1XR1*	*TCF3*	*TERC*
TERT	*TET1*	*TET2*	*TMEM30A*	*TMSB4X*	*TNFAIP3*
TNFRSF14	*TOX*	*TP53*	*TPMT*	*TRAF3*	*U2AF1*
UBE2A	*UBR5*	*USP7*	*VAV1*	*VHL*	*WHSC1*

WT1	*XBP1*	*XPO1*	*ZAP70*	*ZBTB7A*	*ZFP36L1*
ZMYM3	*ZNF292*	*ZRSR2*			

Supplemental Table 4. Clinical characteristics and comparison of different stages of PMF patients

Clinical characteristics	Pre-PMF(N=69)	*P*1	Overt-PMF(N=161)	*P*2	PMF-AP/BP(N=45)	*P*3
Male (%)	34（49.3）	0.474	88（54.7）	0.521	23（51.1）	0.848
Age (years,median,range)	56（8 ~ 85）	0.672	55（11 ~ 77）	0.039	60（33 ~ 75）	0.31
HGB (median,range, g/L)	133（62 ~ 180）g/L	<0.001	97（24 ~ 169）g/L	0.004	83（36 ~ 122）g/L	<0.001
WBC (median,range, x 10^9/L)	10.88(1.73 ~ 30.57)	0.007	8.1(0.45 ~ 139.7)	0.005	14.45(1.34 ~ 144.38)	0.102
PLT(median,range, x 10^9/L)	935(20 ~ 2562)	<0.001	232(1 ~ 1921)	0.003	83(2 ~ 892)	<0.001
ALB (median,range, g/L)	43.1（33.5 ~ 50.6）	0.009	41.9（28.3 ~ 53.3）	<0.001	37.6（28.2 ~ 50.5）	<0.001
LDH (median,range, U/L)	287.2(129.8 ~ 965.6)	<0.001	473.9(124.8 ~ 2410.6)	<0.001	925.95(204.1 ~ 3150.1)	<0.001
> Normal range,n(%)	41(68.3%)	0.009	124(84.4%)	0.074	40(95.2%)	0.001
PB blast(median,range,%)	0(0 ~ 2)	<0.001	0（0 ~ 9）	<0.001	15（1 ~ 81）	<0.001
BM blast (median,range,%)	0(0 ~ 3.5)	0.003	0.5（0 ~ 8）	<0.001	21（2.5 ~ 69）	<0.001
TNF-a (median,range,pg/ml)	24.05（6.2 ~ 56.8）	0.233	28.45（5.46 ~ 451）	0.003	66.2（12.7 ~ 450）	0.003
IL-1β（median,range, pg/ml）	5(5 ~ 85.3)	0.098	5(5 ~ 43.5)	0.038	6.54(5 ~ 18.3)	1.000
IL-2 receptor（median,range, U/ml）	607(96.5 ~ 1207)	0.090	737(215 ~ 4110)	0.015	953.5(430 ~ 7500)	0.004
IL-6 (median,range, pg/ml)	4.58(2 ~ 144)	0.269	7.86(2 ~ 45.2)	0.079	13.45(2 ~ 92)	0.110
IL-8 (median,range, pg/ml)	378(10.2 ~ 3517)	0.071	100.35(5.8 ~ 2391)	<0.001	571.5(26.5 ~ 7500)	0.204
IL-10 (median,range, pg/ml)	5(5 ~ 5.79)	0.669	5(5 ~ 100)	0.028	5(5 ~ 46.8)	0.070
Splenomegaly (n,%)	19（27.9%）	<0.001	113（72%）	0.304	32（80%）	<0.001
Transfusion dependence (n,%)	0（0%）	<0.001	46（28.6%）	0.149	18（40%）	<0.001
Constitutional symptom (n,%)	8（11.6%）	0.023	40（24.8%）	<0.001	24（53.3%）	<0.001
Unfavorable karyotype (n,%) (MIPSS70-plus)	8（14.3%）	0.177	29（23%）	<0.001	23（60.5%）	<0.001

*P*1 Pre-PMF *vs.* Overt-PMF ; *P*2 Overt-PMF *vs.* PMF-AP/BP; *P*3 Pre-PMF *vs.* PMF-AP/BP

Abbreviations: HGB: hemoglobin; WBC: white blood cell; PLT: blood platelet; ALB:albumin; LDH：lactic dehydrogenase; PB:Peripheral blood; BM:bone marrow;TNF-a: tumor necrosis factor-a;IL-1β:interleukin-1β;IL-2 : interleukin-2;IL-6: interleukin-6;IL-8: interleukin-8;IL-10: interleukin-10;

MIPSS70-plus: Development and Validation of the Cytogenetic Enhanced mutation-enhanced international prognostic score system for transplantation-age patients.

Supplemental Table 5. Univariate and multivariate analysis of enrichment of non-driver mutations in different stages of PMF.

Overt-PMF *vs.* PMF-AP/BP						
	Univariate analysis			Multivariate analysis		
Gene	Odds ratio	95%CI	*P* value	Odds ratio	95%CI	*P* value
ASXL1	2.32	1.18~4.55	0.013	2.88	1.36~6.10	0.006
SETBP1	2.69	1.02~7.64	0.039	2.35	0.81~6.81	0.114
RUNX1	4.83	1.82~12.76	0.001	5.11	1.66~15.74	0.004
NRAS	5.75	1.73~19.10	0.002	4.06	1.05~15.60	0.042
SRSF2	2.89	1.27~6.58	0.009	1.87	0.73~4.79	0.196
Pre-PMF *vs.* Overt-PMF						
	Univariate analysis			Multivariate analysis		
Gene	Odds ratio	95%CI	*P* value	Odds ratio	95%CI	*P* value
ASXL1	3.99	1.71~9.33	0.001	3.65	1.54~8.62	0.003
U2AF1	10.76	1.42~81.53	0.005	9.21	1.20~70.69	0.033

Supplemental Table 6. The mutations of 17 paired serial samples from PMF patients.

Patient	Overt-PMF phase	VAF	Accelerate/Blast phase	VAF
UPN688	*JAK2V617F*	91.4	*JAK2V617F*	95.8
	ASXL1	45.3	*ASXL1*	45.5
UPN809	*JAK2V617F*	14.2	*JAK2V617F*	17
	ASXL1	11.8	*ASXL1*	17.7
	TET2	4.2	*TET2*	2.4
	NF1 p.Q1724X	11.6	*NF1 p.Q1703X*	19.6
	DDX41	23.2	*DDX41*	15.5
	ETNK1	5.7	*ETNK1*	11.7
	EZH2	11.4	*EZH2*	23.6
			*ETV6 p.S139Yfs*14*	2.4
			ETV6 p.R369W	12.4
			SETBP1	1.8
			TP53	11.1
			TET2 p.F1287S	2.2
UPN236	*JAK2V617F*	45.4	*JAK2V617F*	37.31
	ASXL1	43.6	*ASXL1*	34.41
	IDH2	47.8	*SRSF2*	2
	SRSF2	45.5	*RUNX1*	15.91
	BRIP1	48.5	*NOTCH2*	45.59
UPN045	*CALR*	50	*CALR*	14
	ASXL1	5.7	*ASXL1*	2.3
			NF1	32.3
UPN927	*CALR*	49.7	*CALR*	38.9
	ASXL1	41.9	*ASXL1*	44.5
	SETBP1 p.D868N	12.81	*SETBP1 p.D868N*	43.4
	EZH2	87	*EZH2*	81
	SETBP1 p.D868Y	26.5	*ETV6 p.N85Kfs*5*	41.4
	ETV6 p.R105X	28.7	*NRAS*	40.6
UPN578	*CALR*	48.4	*CALR*	14.8
	SF3B1 p.K700E	14.7	*SF3B1 p.K700E*	34
	SF3B1 p.K666N	4.7	*SF3B1 p.K666N*	3.5
UPN546	*MPLW515K*	50	*MPLW515K*	50
	SETBP1	44.97	*SETBP1*	48.8
	SUZ12	40.31	*SUZ12*	55.4

			NRAS p.G12V	8.8
			NRAS p.G12D	8.7
			NRAS p.G12A	26.8
			SRSF2	58.1
			RUNX1	16.7
			BCOR	48.9
UPN834	*CALR*	40	*CALR*	16.4
	PLCG1	39.1	*PLCG1*	9.7
	FAT1	49.8	*CBL*	70.2
			CSMD1	2.9
			RUNX1	60.8
UPN853	*JAK2V617F*	6.82	*JAK2V617F*	35.8
	CCND3	5.6	*CCND3*	31
	SETBP1	47.16	*RUNX1*	38.6
UPN511	*MPLW515K*	50	*MPLW515K*	50
	SRSF2	51.4	*SRSF2*	45.8
			ETV6	12.2
			DIS3	45.3
UPN705	*JAK2V617F*	80.2	*JAK2V617F*	94.6
	SRSF2	48.9	*SRSF2*	44.8
	RUNX1	47	*RUNX1*	49.1
	TET2	5.5		
UPN667	*JAK2V617F*	44.5	*JAK2V617F*	56
	SF3B1	27.9	*SF3B1*	33.1
			ASXL1	41.3
			GNAS	37.1
			SETD1B	39.9
UPN663	*JAK2V617F*	2.1	*JAK2V617F*	43.5
	SRSF2	52.2	*SRSF2*	48.4
			ASXL1	31.4
**Patient**	**Pre-PMF phase**	**VAF**	**Overt-PMF phase**	**VAF**
UPN490	*JAK2V617F*	39.9	*JAK2V617F*	36.9
	DNMT3A	48.7	*DNMT3A*	39.7
	SF3B1	47.4	*SF3B1*	42.7
	TP53 p.K132R	5	*TP53 p.K132R*	1.6
	KMT2D	50.6	*TP53 p.E258K*	2.2
	FGFR3	46.9		
	NOTCH1	36.2		
UPN755	*ATG2B*	47.4		
UPN127	*ZMYM3*	50.3	*ZMYM3*	52.6
UPN312	*PRF1*	45.7		
	PDGFRB	50.9		

Reference

[1] Li B, Gale RP, Xu ZF, Qin TJ, Song Z, Zhang PH, et al. Non-driver mutations in myeloproliferative neoplasm-associated myelofibrosis. *J Hematol Oncol*. 2017;10(1):99
